# Breast cancer stage at diagnosis and area-based socioeconomic status: a multicenter 10-year retrospective clinical epidemiological study in China

**DOI:** 10.1186/1471-2407-12-122

**Published:** 2012-03-29

**Authors:** Qiong Wang, Jing Li, Shan Zheng, Jia-Yuan Li, Yi Pang, Rong Huang, Bao-Ning Zhang, Bin Zhang, Hong-Jian Yang, Xiao-Ming Xie, Zhong-Hua Tang, Hui Li, Jian-Jun He, Jin-Hu Fan, You-Lin Qiao

**Affiliations:** 1 Department of Epidemiology, West China School of Public Health, Sichuan University, 16 Ren Min Nan Lu, Chengdu, Sichuan 610041, China; 2 Department of Cancer Epidemiology, Cancer Institute & Hospital, Chinese Academy of Medical Sciences & Peking Union Medical College, 17 South Panjiayuan Lane, Beijing 100021, China; 3 Department of Pathology, Cancer Institute & Hospital, Chinese Academy of Medical Sciences & Peking Union Medical College, 17 South Panjiayuan Lane, Beijing 100021, China; 4 Center of Breast Disease, Cancer Institute & Hospital, Chinese Academy of Medical Sciences & Peking Union Medical College, 17 South Panjiayuan Lane, Beijing 100021, China; 5 Department of Breast Surgery, Liaoning Cancer Hospital, 44 Xiaoyanhe Road, Dadong District, Shenyang 110042, China; 6 Department of Breast Surgery, Zhejiang Cancer Hospital, 38 Banshanqiao Guanji Road, Hangzhou 310022, China; 7 Department of Breast Oncology, Sun Yat-Sen University Cancer Center, 651 Dongfeng East, Gungzhou 510060, China; 8 Department of Breast-thyroid Surgery, Xiangya Sencod Hospital, Central South University, 139 Renminzhonglu, Changsha 410011, China; 9 Department of Breast Surgery, the Second People's Hospital of Sichuan Province, Chengdu 610041, China; 10 Department of Oncosurgery, the First Affiliated Hospital of Medical College, Xi'an Jiao Tong University, 277 Yanta West Road, Xi'an 710061, China

**Keywords:** Breast cancer, Stage at diagnosis, Area-based socioeconomic status, Nation-wide, Multi-center, Retrospective study

## Abstract

**Background:**

Although socioeconomic status (SES) has been focused on as a key determinant of cancer stage at diagosis in western countries, there has been no systemic study on the relationship of SES and breast cancer stage at diagnosis in China.

**Methods:**

The medical charts of 4,211 eligible breast cancer patients from 7 areas across China who were diagnosed between 1999 and 2008 were reviewed. Four area-based socioeconomic indicators were used to calculate area-based SES by cluster analysis. The associations between area-based SES and stage at diagnosis were analyzed by trend chi-square tests. Binary logistic regression was performed to estimate odds ratios for individual demographic characteristics' effects on cancer stages, stratified by area-based SES.

**Results:**

The individual demographic and pathologic characteristics of breast cancer cases were significantly different among the seven areas studied. More breast cancer cases in low SES areas (25.5%) were diagnosed later (stages III & IV) than those in high (20.4%) or highest (14.8%) SES areas (*χ*^2 ^for trend = 80.79, *P *< 0.001). When area-based SES is controlled for, in high SES areas, cases with less education were more likely to be diagnosed at later stages compared with more educated cases. In low SES areas, working women appeared to be diagnosed at earlier breast cancer stages than were homemakers (OR: 0.18-0.26).

**Conclusions:**

In China, women in low SES areas are more likely to be diagnosed at later breast cancer stages than those in high SES areas.

## Background

Breast cancer is by far the most common cancer among women both in developed and developing regions, with an estimated 1.38 million new cancer cases diagnosed worldwide in 2008 (23% of all cancers) [1]. In recent years, both incidence of and mortality from breast cancer have declined in the United States; between 1999 and 2006, incidence rates decreased by 2.0% per year, and mortality decreased by 1.9% annually between 1998 and 2006 [2]. However, the incidence of breast cancer is steadily rising in developing countries [3-6]. In China, the incidence of breast cancer rose from 126,227 cases in 2002 [7] to over 169,000 in 2008 [1].

Preventive screening for breast cancer can increase the number of individuals diagnosed at an early stages and reduce mortality. In China, the main methods of breast cancer screening are clinical breast examination (CBE), mammography, high-frequency ultrasound, and magnetic resonance imaging (MRI). However, due to the lack of large population-based screening evaluations in China, there is no consistent evidence indicating which screening method or combination of methods is best for the country. To date, nationwide breast cancer screening has never been implemented routinely in China, and in places where routine breast cancer screening exists, its usage varies with area-based socioeconomic status (SES) [8,9]. Individuals living in poorer areas are less likely to seek cancer screening compared with individuals living in wealthier areas [10]. Studies over the past several decades have indicated that individuals living in less developed areas often had worse general health compared with individuals living in relatively developed areas [11-17]. The majority of studies investigating associations between SES and breast cancer stage at diagnosis have also documented socioeconomic and geographic disparities, with higher incidence of late stage breast cancer in lower income areas [18-24]. Regional disparities in SES are significant in China, where economic development in eastern cities generally started earlier and has been faster than that in the country's interior and in rural areas [25].

Breast cancer survival rates decline with delayed diagnosis. The Surveillance Epidemiology and End Results (SEER) showed that 98.3% of women treated for early-stage breast disease survive five or more years, while only 83.5% of women diagnosed with regional breast cancer and 23.3% of those with distant breast cancer survive beyond five years of the initial diagnosis [2]. Thus, as improvement in breast cancer prognosis would have a significant impact on countries' health budgets, and SES disparities result in different breast cancer outcomes, strategies are urgently needed that target higher-risk areas and resonate with higher-risk population subgroups. Breast cancer prevention and control has been prioritized by China's Ministry of Health, but so far, there has been no systemic study of the relationship between SES and breast cancer stage in the country. In this study, based on the Nationwide Multicenter 10-year (1999-2008) Retrospective Clinical Epidemiological Study of Breast Cancer in China, directed by the Cancer Hospital/Institute, Chinese Academy of Medical Sciences (CICAMS), we explored the effects of both area level factors, namely SES, and individual demographic characteristics on breast cancer stage at diagnosis.

## Methods

### Hospital selection, case sampling, and data collection

Hospital selection, case sampling, and data collection methods have been previously described in detail [26]. Briefly, all of China was stratified into seven geographic areas (north, northeast, central, south, east, northwest, and southwest). Then, purposive sampling was used to choose one tertiary public cancer hospital in each region with the following characteristics. Firstly, participant hospitals were the leading public cancer hospitals and regional referral centers providing pathology diagnosis, surgery, radiotherapy, medical oncology, and routine follow-up care for patients with breast cancer. Secondly, their patient source had to include the entire study region. Finally, since patients tend to seek better medical service in big cities, the hospitals were each located in a major city. The participant hospitals were the Cancer Institute & Hospital, Chinese Academy of Medical Sciences (in Beijing city, north China), Liaoning Cancer Hospital (in Liaoning Province, northeast China), Second Xiangya Hospital, Central South University (in Hunan Province, central China), Sun Yat-Sen University Cancer Center (in Guangdong Province, south China), Zhejiang Cancer Hospital (in Zhejiang Province, east China), First Affiliated Hospital of Xi'an Jiaotong University (in Shannxi Province, northwest China), and Sichuan Cancer Hospital (in Sichuan Province, southwest China).

The selected hospitals provided us with the medical records of breast cancer patients diagnosed between 1999 and 2008. For each year in each hospital, one month was randomly selected, and all inpatient cases for that month were reviewed (January and February were excluded from the random selection to eliminate any confounding effects of China's largest annual holiday). The hospital records were reviewed in each local hospital by local clerks who had been trained systematically in Beijing. Then, standard case report forms (CRF) designed by CICAMS [26] was used to extract the primary medical reports of every qualified patient, including general information, risk factors, diagnostic imaging tests, therapy models, and pathologic characteristics. The reliability and validity of CRF had been assessed by proceeding a pilot study and CRF was regarded as reliable and valid. After collection, the raw variables were coded and sorted into different categories for analysis. Cancer stage at diagnosis was categorized into six groups by archiaters referring to pathologic reports and using the American Joint Committee on Cancer (AJCC) TNM System (0, I, II, III, IV, and unknown stage or not applicable). The cases admitted between 1999 and 2002 were staged using the fifth version (revised in 1997) [27], and the cases from 2003-2008 were staged with the sixth version (revised in 2002) [28]. If individuals were diagnosed with more than one primary breast tumor at the same time, we included only the record of the tumor at the most advanced stage. We excluded tumors with an unknown or not applicable stage, and grouped the breast cancer stages of the other cases into two categories: "early" (stages 0 & I) or "non-early" (stages II & III & IV).

The study protocol was approved by the Cancer Foundation of China Institutional Review Board and data were stripped of all personal identifiers, per the board's approved procedures.

### Area-based socioeconomic indicators

Area-based SES measures derived from census data have been shown to be related to various health outcomes independent of individual SES [11,12,22,29,30]. Because there is a lack of accepted SES measures in China, we referred to the socioeconomic measures of "percent of the population living below poverty line" and "percent of the population ≥ 25 years of age without a high school diploma" in the United States [31] to devise our own socioeconomic indicators (SEIs) reflecting the economic and education status of the seven areas. These are "GDP per capita," "percentage of health-service expenditure in the regional/provincial public affairs general budget (HSE percentage)," "ratio of urban to rural population (PU/PR ratio)," and "percentage of illiteracy in females aged 15 and over (FPI percentage)." The SEIs of each district or province with a selected hospital were obtained from annual reports issued by the National Bureau of Statistics of the People's Republic of China from 1999 to 2008, except that data on the PU/PR ratio was available only from 2005 to 2008. These SEIs were used to represent the SES of each area.

### Data Analysis

Descriptive statistics were used to summarize the demographic and pathologic characteristics of the study population. The mean and standard deviation of quantitative variables were calculated, and the differences among the seven areas were analyzed with one-way ANOVA. Differences in distribution of variables were examined using Mantel-Haenszel chi-square tests or Fisher's exact tests.

The single SEI values among the seven areas were compared using one-way ANOVA followed by the Student-Newman-Keuls (SNK) test, a post hoc tests that measures significance among multiple groups. Those areas between which there were no statistically significant differences were combined, forming ordinal categorical levels of SEI status. Trend chi-square tests were used to examine associations between stage at diagnosis and the SEIs status. Additionally, area-based SES was calculated by cluster analysis, an exploratory data analysis tool which sorts objects into groups such that the degree of association between two objects is maximal if they belong to the same group and minimal otherwise. During cluster analysis, the k-means clustering was applied with k = 3, so that the seven regions were classified into three levels based on four SEIs. Binary logistic regression was performed stratified by area-based SES to further explore the association between individual demographic characteristics and breast cancer stage at diagnosis (non-early stage vs. early stage) in SES subgroups. SPSS statistical software version 17.0 was used to analyze the data. Statistical significance was assessed by two-tailed tests with an α level of 0.05.

## Results

### SEIs, individual demographic and pathologic characteristics by area

A total of 4,211 eligible cases were included in our study. In general, the differences in SEIs among the seven areas were significant (*P *< 0.001) (Table 1). During the period studied, the mean of GDP per capita across the seven areas was 13,048 ± 5,567 yuan per person, ranging from 8,050 ± 3,599 in the southwest to 36,146 ± 17,217 in northern China. More than 70% of breast cancer patients were 40-69 years old when diagnosed. Cases in the north (42.1%), northeast (32.8%), and south (29.8%) were more likely to be overweight at diagnosis (BMI ≥ 25.00 kg/m^2^) than were those in other areas (3.9%-18.8%). Women in eastern, northwestern, and southwestern China were mainly occupied in manual work (80.4%, 54.0%, and 62.7%, respectively), while more women were homemakers in central China (13.2%) than in other areas (average across all areas = 4.0%). Information on patients' education levels was generally unavailable in north, northeast, and southwest China. Among the other four areas, we found that women in central China had higher education levels, with 27.5% receiving higher education (university and above). The vast majority of cases in all seven areas were married. All the above demographic characteristics of breast cancer cases were significantly different among the seven areas (Table 2, *P *< 0.001).

**Table 1:** SEIs of seven regions

Variable by level	Total (Range)	Beijing (North)	Liaoning (Northeast)	Hunan (Central)	Guangdong (South)	Zhejiang (East)	Shannxi (Northwest)	Sichuan (Southwest)	***F ***(***P*-value**)	SNK* (No. of subgroups)
SEI										
GDP per capita^(1)^	13048 ± 5567 (8050-36146)	36146 ± 17217^a^	17200 ± 6762^b^	9223 ± 4019^c^	21292 ± 9165^b^	23832 ± 10331^b^	8840 ± 4615^c^	8050 ± 3599^c^	12.69 (< 0.001)	3
HSE percentage^(2)^	4.47 ± 0.43 (3.18-6.51)	6.51 ± 0.51^a^	3.18 ± 0.41^c^	3.41 ± 0.74^c^	4.27 ± 0.45^b^	5.71 ± 0.71^a^	3.87 ± 0.75^c^	4.56 ± 0.66^b^	38.69 (< 0.001)	3
PU/PR ratio^(3)^	0.80 ± 0.04 (0.54-5.39)	5.39 ± 0.22^a^	1.45 ± 0.03^c^	0.66 ± 0.06^d^	1.67 ± 0.09^b^	1.32 ± 0.04^c^	0.66 ± 0.06^d^	0.54 ± 0.05^d^	1210 (< 0.001)	4
FPI percentage^(4)^	15.17 ± 2.98 (7.20-17.74)	7.21 ± 1.63^a^	7.20 ± 1.60^a^	11.31 ± 2.60^b^	9.89 ± 2.57^b^	17.30 ± 3.07^c^	15.86 ± 4.30^c^	17.74 ± 3.31^c^	23.02 (< 0.001)	3

*: The single SEI values among the seven areas were compared using one-way ANOVA followed by the Student-Newman-Keuls (SNK) test, a post hoc test for significant differences among multiple groups. The areas between which there were no statistically significant differences were combined to form subgroups. a, b, c, and d represent different subgroups for each variable; if two or more regions are followed by the same letter, there is no significant difference between them.

^(1)^: yuan per person per year (mean ± SD, 1999-2008)

^(2)^: percentage of health-services expenditure in the regional/provincial public affairs general budget (%) (mean ± SD, 1999-2008)

^(3)^: ratio of urban to rural population (mean ± SD, 2005-2008) (data is available in the National Bureau of Statistics of the People's Republic of China only for 2005 to 2008)

^(4)^: percentage of illiteracy among women aged 15 and over (%) (mean ± SD, 1999-2008)

**Table 2:** Individual demographic characteristics by region

	Total	Beijing (North)	Liaoning (Northeast)	Hunan (Central)	Guangdong (South)	Zhejiang (East)	Shannxi (Northwest)	Sichuan (Southwest)	Statistical value	*P*-value
Demographic Characteristic	Total N = 4211	Total N = 641	Total N = 832	Total N = 546	Total N = 604	Total N = 606	Total N = 483	Total N = 499		
	N (%)	N (%)	N (%)	N (%)	N (%)	N (%)	N (%)	N (%)		
**Age at diagnosis (years)**										
Mean ± SD	48.68 ± 10.47	50.45 ± 10.92	48.88 ± 9.95	48.25 ± 10.34	47.82 ± 10.94	47.35 ± 9.58	50.10 ± 11.01	47.86 ± 10.32	7.63*	< 0.001
≤ 40	919 (21.8)	113 (17.6)	161 (19.4)	133 (24.4)	155 (25.7)	147 (24.3)	89 (18.4)	121 (24.2)		
40-69	3126 (74.2)	489 (76.3)	645 (77.5)	387 (70.9)	429 (71.0)	443 (73.1)	369 (76.4)	364 (72.9)		
≥ 70	166 (3.9)	39 (6.1)	26 (3.1)	26 (4.8)	20 (3.3)	16 (2.6)	25 (5.2)	14 (2.8)		
**Body Mass Index (kg/m**^**2**^**)^(1)^**										
Mean ± SD	23.35 ± 2.89	24.65 ± 3.46	24.01 ± 3.08	21.72 ± 1.85	23.25 ± 3.47	22.28 ± 2.86	23.25 ± 1.29	23.25 ± 1.82	67.42*	< 0.001
≤ 24.99	3287 (78.1)	371 (57.9)	559 (67.2)	520 (95.2)	422 (70.2)	492 (81.2)	464 (96.1)	460 (92.2)		
≥ 25.00	921 (21.9)	270 (42.1)	273 (32.8)	26 (4.8)	180 (29.8)	114 (18.8)	19 (3.9)	39 (7.8)		
**Occupation**										
Homemaker	173 (4.0)	17 (2.7)	2 (0.2)	72 (13.2)	39 (6.5)	11 (1.8)	14 (2.9)	18 (3.6)	6.52**	< 0.001
Manual worker	1893 (45.0)	230 (35.9)	258 (31.1)	185 (33.9)	159 (26.3)	487 (80.4)	261 (54.0)	313 (62.7)		
White-collar worker	1028 (24.4)	203 (31.7)	174 (20.9)	168 (30.8)	167 (27.6)	61 (10.1)	153 (31.7)	102 (20.4)		
Other	529 (12.6)	70 (10.9)	99 (11.9)	114 (20.9)	162 (26.8)	40 (6.6)	19 (3.9)	25 (5.0)		
Unknown	588 (14.0)	121 (18.9)	299 (35.9)	7 (1.2)	77 (12.8)	7 (1.1)	36 (7.5)	41 (8.2)		
**Education**										
None	186 (4.4)	5 (0.8)	1 (0.1)	0 (0.0)	41 (6.8)	124 (20.5)	14 (2.9)	1 (0.2)	4.63***	< 0.001
Primary school	462 (11.0)	3 (0.5)	0 (0.0)	125 (22.9)	116 (19.2)	173 (28.5)	39 (8.1)	6 (1.2)		
Middle school	606 (14.4)	0 (0.00)	0 (0.0)	147 (26.9)	172 (28.5)	165 (27.2)	115 (23.8)	7 (1.4)		
High school	441 (10.5)	0 (0.00)	2 (0.2)	122 (22.3)	143 (23.7)	56 (9.2)	116 (24.0)	2 (0.4)		
University and above	396 (9.4)	4 (0.6)	23 (2.8)	150 (27.5)	112 (18.5)	29 (4.8)	76 (15.7)	2 (0.4)		
Unknown	2120 (50.3)	629 (98.1)	806 (96.9)	2 (0.4)	20 (3.3)	59 (9.8)	123 (25.5)	481 (96.4)		
**Marital Status**										
Single	51 (1.2)	6 (0.9)	9 (1.1)	4 (0.7)	17 (2.8)	5 (0.8)	3 (0.6)	7 (1.4)	56.43**	< 0.001
Married	4090 (97.1)	620 (96.7)	821 (98.7)	525 (96.2)	586 (97.0)	589 (97.2)	474 (98.1)	475 (95.2)		
Widowed/Divorced	52 (1.2)	12 (1.9)	0 (0.0)	17 (3.1)	0 (0.0)	7 (1.2)	5 (1.0)	11 (2.2)		
Unknown	18 (0.5)	3 (0.5)	2 (0.2)	-	1 (0.2)	5 (0.8)	1 (0.2)	6 (1.2)		

*: one-way ANOVA

**: Chi-square test

***: Fisher's exact test

^(1)^: Missing BMI values were replaced with the mean from available data, 23.35 kg/m^2^

Invasive ductal carcinoma was the dominant pathologic subtype, ranging from 70.1% of cases in the northeast to 92.4% in central China. Among 3555 cases that were tested for estrogen receptor (ER) and progesterone receptor (PR), 3534 had test results included in their records. About half of these (47.6%) were ER + and PR+, and 32.0% were ER- and PR-. In northwest China, only 282 (58.4%) cases were tested for ER/PR and 38.7% of them were ER + and PR+; both of these rates were far lower than in the other areas. Human epidermal growth factor receptor 2 (Her-2) information was available for 2849 patients, the majority of whom (2113, 65.0%) were Her-2 negative. The north had a high testing rate (609/641, 95.0%) and the highest positive rate (286/609, 47.0%) for Her-2. All of the pathologic characteristics of breast cancer cases were significantly different across the seven areas (Table 3, *P *< 0.001).

**Table 3:** Individual pathologic characteristics by region

	Total	Beijing (North)	Liaoning (Northeast)	Hunan (Central)	Guangdong (South)	Zhejiang (East)	Shannxi (Northwest)	Sichuan (Southwest)	***χ***2	*P*-value
Pathological Characteristics	Total N = 4211	Total N = 641	Total N = 832	Total N = 546	Total N = 604	Total N = 606	Total N = 483	Total N = 499		
	N (%)	N (%)	N (%)	N (%)	N (%)	N (%)	N (%)	N (%)		
**Type of Postoperative Pathological Diagnosis^(1)^**										
Carcinoma in Situ (CIS)	143 (3.4)	61 (9.5)	22 (2.6)	17 (3.1)	19 (3.1)	7 (1.2)	5 (1.0)	12 (2.4)	169.1	< 0.001
Invasive Ductal Carcinoma	3471 (82.4)	528 (82.4)	583 (70.1)	504 (92.4)	528 (87.4)	523 (86.3)	406 (84.1)	399 (80.0)		
Other Invasive Carcinoma	385 (9.1)	47 (7.3)	87 (10.5)	21 (3.8)	29 (4.8)	59 (9.7)	70 (14.5)	72 (14.4)		
Other	15 (0.4)	5 (0.8)	0 (0.0)	1 (0.2)	4 (0.7)	0 (0.0)	2 (0.4)	3 (0.6)		
Unavailable	197 (4.7)	-	140 (16.8)	3 (0.5)	24 (4.0)	17 (2.8)	-	13 (2.6))		
**Pathological stage^(2)^**										
Stage 0	39 (0.9)	7 (1.1)	13 (1.6)	6 (1.1)	8 (1.3)	4 (0.7)	0 (0.0)	1 (0.2)	450.5	< 0.001
Stage I	663 (15.7)	172 (26.8)	131 (15.7)	73 (13.4)	98 (16.2)	95 (15.7)	77 (15.9)	17 (3.4)		
Stage II	1891 (44.9)	280 (43.7)	323 (38.8)	400 (73.3)	243 (40.2)	239 (39.4)	170 (35.2)	236 (47.3)		
Stage III	788 (18.7)	93 (14.5)	119 (14.3)	53 (9.7)	117 (19.4)	152 (25.1)	127 (26.3)	127 (25.5)		
Stage IV	113 (2.7)	2 (0.3)	4 (0.5)	3 (0.5)	20 (3.4)	5 (0.8)	56 (11.6)	23 (4.6)		
Unavailable	717 (17.1)	87 (13.6)	242 (29.1)	11 (2.0)	118 (19.5)	111 (18.3)	53 (11.0)	95 (19.0)		
**ER/PR test**										
Performed	3555 (84.4)	616 (96.1)	711 (85.5)	535 (98.0)	511 (84.6)	462 (76.2)	282 (58.4)	438 (87.8)	483.2	< 0.001
Not performed	471 (11.2)	24 (3.7)	85 (10.2)	8 (1.5)	54 (8.9)	67 (11.1)	192 (39.8)	41 (8.2)		
Unknown	185 (4.4)	1 (0.2)	36 (4.3)	3 (0.5)	39 (6.5)	77 (12.7)	9 (1.8)	20 (4.0)		
**Her-2 test**										
Performed	3251 (77.2)	609 (95.0)	693 (83.3)	533 (97.6)	469 (77.6)	424 (70.0)	241 (49.9)	282 (56.5)	640.2	< 0.001
Not performed	762 (18.1)	31 (4.8)	98 (11.8)	8 (1.5)	99 (16.4)	98 (16.1)	227 (47.0)	201 (40.3)		
Unknown	198 (4.7)	1 (0.2)	41 (4.9)	5 (0.9)	36 (6.0)	84 (13.9)	15 (3.1)	16 (3.2)		
**ER/PR status^(3)^**										
ER+&PR+	1691 (47.6)	373 (60.6)	318 (44.7)	244 (45.6)	241 (47.2)	221 (47.8)	109 (38.7)	185 (42.2)	121.0	< 0.001
ER+&PR-	337 (9.5)	44 (7.1)	79 (11.1)	54 (10.1)	37 (7.2)	41 (8.9)	31 (11.0)	51 (11.6)		
ER-&PR+	367 (10.3)	47 (7.6)	108 (15.2)	32 (6.0)	75 (14.7)	34 (7.4)	37 (13.1)	34 (7.8)		
ER-&PR-	1139 (32.0)	146 (23.7)	206 (29.0)	203 (37.9)	152 (29.7)	165 (35.7)	101 (35.8)	166 (37.9)		
Unavailable	21 (0.6)	6 (1.0)	-	2 (0.4)	6 (1.2)	1 (0.2)	4 (1.4)	2 (0.5)		
**Her-2 status^(4)^**										
Her-2 +	736 (22.6)	286 (47.0)	36 (5.2)	156 (29.3)	90 (19.2)	68 (16.0)	29 (12.0)	71 (25.2)	339.6	< 0.001
Her-2 -	2113 (65.0)	299 (49.1)	545 (78.6)	314 (58.9)	337 (71.9)	317 (74.8)	181 (75.1)	120 (42.6)		
Uncertain/unavailable	402 (12.4)	24 (3.9)	112 (16.2)	63(11.8)	42 (9.1)	39 (9.2)	31 (12.9)	91 (32.2)		

^(1)^: carcinoma in situ: ductal carcinoma in situ, lobular carcinoma in situ, microinvasive ductal carcinoma, and Paget's disease invasive ductal carcinoma: pure invasive ductal carcinoma and mixed invasive ductal carcinoma other invasive carcinomas: invasive labular carcinoma, tubular carcinoma, medullary carcinoma, mucin carcinoma, and other invasive carcinomas

^(2)^: according to the AJCC/UICC TNM system

^(3)^: ER/PR status was based on all breast cancer cases tested for ER/PR

^(4)^: Her-2 status was based on all breast cancer cases tested for Her-2

### SEIs, area-based SES, and breast cancer stage at diagnosis

For the analysis of factors affecting breast cancer stage at diagnosis, we excluded 717 cases with an unknown or not applicable stage, so that the final number of cases in the following analyses was 3494.

We found an inverse relationship between breast cancer stage and both GDP per capita and ratio of urban to rural population, while percentage of illiteracy in females aged 15 and over was positively related to breast cancer stage (*P *for trend < 0.001) (Table 4). However, there was no significant trend correlation between percentage of health-service expenditure in the regional/provincial public affairs general budget and breast cancer stage (*χ*^2 ^for trend = 3.307, *P *= 0.081). The area-based SES based on the four SEIs was negatively correlated with breast cancer stage: 14.8% (95/641) of cases in the highest SES areas were diagnosed at stages III or IV, while 20.4% (417/2024) of cases in high SES areas and 25.5% (389/1528) in low SES areas were diagnosed at these late stages (*χ*^2 ^for trend = 80.79, *P *< 0.001). Patients in Beijing and Guangzhou, where routine breast cancer screening has been implemented for several years [32,33], were more likely to be diagnosed at stages 0 or I (27.9% and 17.5%) than were patients in other areas (Table 3).

**Table 4:** Incorporated SEIs, area-based SES, and breast cancer stage at diagnosis

Variable by level	Mean ± SD	Included Provinces	Total	Stage 0&I	Stage II	Stage III&IV	Unavailable	Chi-square for trend	*P*-value
				N (%)	N (%)	N (%)	N (%)		
**GDP per capita^(1)^**									
Highest	36146 ± 17217	Beijing	641	179 (27.9)	280 (43.7)	95 (14.8)	87 (13.6)	80.79	< 0.001
High	20774 ± 9006	Liaoning, Guangdong, Zhejiang	2024	349 (17.1)	805 (39.4)	417 (20.4)	471 (23.1)		
Low	8704 ± 3986	Hunan, Shannxi, Sichuan	1528	174 (11.4)	806 (52.7)	389 (25.5)	159 (10.4)		
**HSE percentage^(2)^**									
Highest	6.11 ± 0.73	Beijing, Zhejiang	1247	278 (22.3)	519 (41.6)	252 (20.2)	198 (15.9)	3.307	0.081
High	4.42 ± 0.57	Guangdong, Sichuan	1103	124 (11.2)	479 (43.5)	287 (26.0)	213 (19.3)		
Low	3.49 ± 0.70	Liaoning, Hunan, Shannxi	1861	300 (16.1)	893 (48.0)	362 (19.5)	306 (16.4)		
**PU/PR ratio^(3)^**									
Highest	5.39 ± 0.22	Beijing	641	179 (27.9)	280 (43.7)	95 (14.8)	87 (13.6)	72.00	< 0.001
Higher	1.67 ± 0.09	Guangdong	604	106 (17.5)	243 (40.2)	137 (22.7)	118 (19.6)		
High	1.39 ± 0.08	Liaoning, Zhejiang	1438	243 (16.9)	562 (39.1)	280 (19.5)	353 (24.5)		
Low	0.62 ± 0.08	Hunan, Shannxi, Sichuan	1528	174 (11.4)	806 (52.7)	389 (25.5)	159 (10.4)		
**FPI percentage **^(4)^									
Highest	16.97 ± 3.55	Zhejiang, Shannxi, Sichuan	1588	194 (12.2)	645 (40.6)	490 (30.9)	259 (16.3)	135.83	< 0.001
High	10.60 ± 2.62	Hunan, Guangdong	1150	185 (16.1)	643 (55.9)	193 (16.8)	129 (11.2)		
Low	7.21 ± 1.57	Beijing, Liaoning,	1473	323 (21.9)	603 (40.9)	218 (14.8)	329 (22.4)		
**Area-based SES**^(5)^									
Highest	-	Beijing	641	179 (27.9)	280 (43.7)	95 (14.8)	87 (13.6)	80.79	< 0.001
High	-	Liaoning, Guangdong, Zhejiang	2024	349 (17.1)	805 (39.4)	417 (20.4)	471 (23.1)		
Low	-	Hunan, Shannxi, Sichuan	1528	174 (11.4)	806 (52.7)	389 (25.5)	159 (10.4)		

^(1)^: GDP per capita: yuan per person per year (mean ± SD, 1999-2008)

^(2)^: percentage of health-services expenditure in the regional/province public affairs general budget (%) (mean ± SD, 1999-2008)

^(3)^: ratio of urban to rural population (mean ± SD, 2005-2008) (data is available in the National Bureau of Statistics of the People's Republic of China only for 2005 to 2008)

^(4)^: percentage of illiteracy among women aged 15 and over (%) (mean ± SD, 1999-2008)

^(5)^: Area-based SES is a composite measure synthesized by cluster analysis of the four SEIs

### Association between individual demographic characteristics and breast cancer stage at diagnosis stratified by area-based SES

With the exception of percentage of health-service expenditure in the regional/provincial public affairs general budget, each of the SEIs was associated with breast cancer stage (Table 4). To further explore the association between individual demographic characteristics and breast cancer stage at diagnosis, we used binary logistic regression and implemented stratified analysis by area-based SES (Table 5). In north China, the only area categorized as highest SES, education information was unavailable for 50.3% of the cases, so we were unable to analyze the effect of individual demographic characteristics in this subgroup. In high SES areas, education appeared to influence the cancer stage at diagnosis. Compared with the patients who had attended university, those only attended middle and/or high school had about a three-fold higher risk of being diagnosed at a late stage. In low SES areas, working women (manual, white-collar, or other) were less likely to be diagnosed at late breast cancer stages than were homemakers (OR: 0.18-0.66). Other demographic characteristics, including age at diagnosis and marital status, were not associated with breast cancer stage at diagnosis.

**Table 5:** Multivariable logistic regression for association between individual demographic characteristics and breast cancer stage at diagnosis (stage 0 & I, stage II, III & IV), stratified by area-based SES*

Variable	High SES areas		Low SES areas	
	OR (95%CI)	Wald (*P*)	OR (95%CI)	Wald (*P*)
**Age at Diagnosis (Years)**				
≤ 40	1.00		1.00	
40-69	0.94 (0.29-3.09)	0.01 (0.92)	1.18 (0.43-3.23)	0.10 (0.75)
≥ 70	1.02 (0.70-1.51)	0.02 (0.90)	1.40 (0.85-2.29)	1.75 (0.19)
**Body Mass Index (kg/m^2^)**				
≤ 24.99	1.00		1.00	
≥ 25.00	1.37 (0.92-2.06)	2.39 (0.12)	1.09 (0.40-12.97)	0.03 (0.86)
**Occupation**				
Homemaker	1.00		1.00	
Manual worker	0.81 (0.33-2.02)	0.20 (0.65)	0.18 (0.05-0.64)	**6.99 (0.01)**
White-collar worker	0.77 (0.33-1.80)	0.37 (0.54)	0.26 (0.08-0.88)	**4.66 (0.03)**
Other	1.21 (0.45-3.25)	0.14 (0.70)	0.18 (0.05-0.65)	**6.85 (0.01)**
**Education**				
University and above	1.00		1.00	
High school	2.89 (1.30-6.44)	**6.76 (0.01)**	0.42 (0.07-2.36)	0.97 (0.32)
Middle school	2.77 (132-5.79)	**7.27 (0.01)**	0.97 (0.42-2.21)	0.01 (0.94)
Primary school	1.87 (0.99-3.64)	3.43 (0.06)	0.98 (0.47-2.05)	0.01 (0.96)
None	1.64 (0.87-3.08)	2.34 (0.13)	0.74 (0.40-1.36)	0.93 (0.34)
**Marital Status**				
Married	1.00		1.00	
Single	6.27 (0.81-48.76)	3.08 (0.08)	1.01 (0.11-9.51)	0.001 (0.99)
Widowed/Divorced	1.18 (0.14-10.06)	0.02 (0.88)	0.49 (0.15-1.62)	1.38 (0.24)
**ER/PR status**				
ER+&PR+	1.00		1.00	
ER+&PR-	0.97 (0.50-1.90)	0.01 (0.93)	1.10 (0.54-2.23)	0.07 (0.79)
ER-&PR+	1.57 (0.83-2.95)	1.96 (0.16)	0.73 (0.36-1.51)	0.70 (0.40)
ER-&PR-	1.04 (0.69-1.56)	0.04 (0.85)	1.31 (0.81-2.11)	1.19 (0.28)

*: Education information for 50.3% of cases in the highest SES area was unknown, so we did not analyze the effect of individual demographic characteristics in this subgroup

## Discussion

Increasing social inequalities in health in China, coupled with growing inequalities in income and wealth, have focused attention on social class as a key determinant of population health. Our study focused on breast cancer stage at diagnosis as an indicator of health care access and quality, and we found a significant relationship between cancer stage and area-based SES. To our knowledge, ours is the first study to tie SES to health status within a developing country.

In this nationwide multicenter study of breast cancer in China, area-based SES was categorized into three levels: highest, high, and low. The Beijing district in north China was the only highest SES area, and also had the highest GDP per capita, percentage of health-service expenditure in the regional/provincial public affairs general budget, and ratio of urban to rural population, and the lowest percentage of illiteracy in females aged 15 and over. Northeast, south, and east China were high SES areas, while central, northwest, and southwest China were low SES areas. This classification accords with the pattern of economic growth and regional inequality in China during the reform era [25]. We found that cases living in low SES areas were more likely to be diagnosed at a later breast cancer stage than were those in high SES areas.

Exactly how area-based socioeconomic conditions influence the stage at which an individual is diagnosed with breast cancer is complex. One explanatory factor is that individuals residing in areas with high GDP per capita are likely to have more personal income compared with those in low GDP per capita areas. In the United States, lack of ability to pay for screening services is often implicated as the reason why individuals with low incomes, or those living in the poorest areas, have lower screening rates and are more likely to be diagnosed with cancer at a late stage [34]. In the present study, we observed that the patients from hospitals located in Beijing and Guangdong were diagnosed at an earlier stage than others. This may partly be explained by the routine breast cancer screening programs that have been implemented in these two cities for several years [32,33]. However, the screening programs in a city may not reflect the screening situation of the whole region. To evaluate the effect of routine screening on early diagnosis, a comprehensive prospective study is needed to compare districts with and without screening programs.

Local governments with a high percentage of health-service expenditure in their regional/provincial public affairs general budgets (HSE percentage) may provide better medical services, giving residents more access to health care such as breast cancer screening, diagnosis, and treatment, which in turn results in earlier diagnosis. Barrya and Breen found that residence in economically and socially distressed or medically underserved neighborhoods tends to increase the likelihood of late-stage cancer diagnoses [19]. However, we observed no clear trend association between HSE percentage and breast cancer stage at diagnosis. HSE percentage may not be a direct indicator of the level of medical service of an area, since HSE percentage may be inconsistent with an area's economic development. For instance, while northeast China had high GDP per capita, it was the lowest HSE percentage of the areas studied.

In contrast, we found an association between earlier breast cancer stage at diagnosis and higher ratio of urban to rural population (PU/PR ratio). In China, there is wide SES gap between cities and rural areas, and so urban/rural status is highly correlated with SES. A higher PU/PR ratio hints that more individuals in the area live in cities, and thus may have more access to medical services and ability to afford medical insurance than do residents of rural areas. This would in turn make them more likely to seek prompt medical care for any health problems. These differences may contribute to disparities in breast cancer stage at diagnosis. Several studies have shown that patients with health insurance are less likely to be diagnosed at a late stage of breast cancer [22,35], an effect similar to that found for other cancers, such as colorectal cancer [34,36].

In this study, area-based education was measured as percentage of illiteracy in females aged 15 and over (FPI percentage), emphasizing education of females. We found a trend association between FPI percentage and stage at diagnosis: the higher the FPI percentage in an area, the later the stage of breast cancer at which women in the area were diagnosed. After stratified analysis by area-based SES, individual education level appeared to influence the stage at diagnosis in high SES areas, while in low SES areas, working women were less likely to be diagnosed at late breast cancer stages than were homemakers. These results highlight the importance of education in high SES areas. Education may be a proxy variable representing individual knowledge and behaviors toward prevention and screening of breast cancer. Women with lower education level may also have higher risk of alcohol-related death and diseases [37]. Low education itself may also create barriers to receiving recommended screening, since low health literacy, low general literacy, and language barriers impact an individual's ability to navigate the medical service system, understand screening options and recommendations, and communicate with healthcare professionals [38]. Working women in low SES areas were more likely to be diagnosed at early stages, which may result from their high breast cancer screening attendance relative to that of homemakers. The results from our previous study [39] and a study by Damiani et al. [40] show that education and occupation were positively associated with breast cancer screening attendance, which in turn may influence breast cancer stage at diagnosis.

However, after adjusting for area-based SES, we found no significant associations between other individual demographic characteristics and breast cancer stage at diagnosis. Thus, area-based SES appeared to be the main, underlying factor influencing breast cancer stage at diagnosis. Baquet et al. similarly found that SES predicted the likelihood of a group's access to education, certain occupations, and health insurance, as well as income level and living conditions, all of which are associated with a person's chances of being diagnosed with late stage disease and of surviving cancer [41]. Moreover, Singh et al. [42] and Launay et al. [43] found that at every stage of diagnosis, breast cancer patients from lower-income areas had lower 5-year relative survival rates did than did those from higher-income areas. The presence of additional illnesses and treatment disparities may contribute to these differences [44,45]. Compared with less developed areas, more developed areas possess better detection techniques, and their cases have the ability to pay more for more accurate tests and more effective therapy. For instance, we found that in Beijing, the highest SES area, more than 95% of cases were tested for ER/PR/Her-2. In most of the low SES areas, however, only about half of cases were tested for Her-2. Even so, this pattern was in agreement with the guidelines of the Breast Health Global Initiative (BHGI), which contend that Her-2 measurement is problematic in limited-resource settings due to the high cost of immunohistochemical analysis, fluorescence in situ hybridization, and trastuzumab therapy, and recommend introducing this test only at the maximal-resource level [46].

The present study is the first geographically epidemiologic study of breast cancer in China. Although we only included women with breast cancer attended to these 7 regional referral hospitals, we expect the effect of SES and later stage of diagnosis will be much stronger than we found in this study (if we included those women attended at local hospitals, such county hospitals). Our findings thus provide a baseline for understanding the country's patient and tumor characteristics. Our evaluation of associations between breast cancer stage and area-based SES, as well as individual demographic characteristics stratified by area-based SES, may help to identify high risk areas and the most important and controllable regional and individual factors influencing breast cancer stage at diagnosis. However, this was an ecological study, so we must recognize the possibility of ecological fallacy. In addition to the individual-level variables examined in this study, it would be useful to examine the effects of other variables, such as access to breast cancer screening services, alcohol use, family history of breast cancer, and lack of exercise, as they may affect breast cancer stage at diagnosis by influencing women's attitudes and behaviour to breast cancer prevention and screening. However, their effects could not be analyzed in this study due to the high proportion of missing data; we plan to test the effects of these variables on breast cancer stage in a future study. Another limitation is that we lacked data on individual SES, which was likely strongly correlated with both education level and area-based SES. Although our method of calculating area-based SES may not have assessed actual socioeconomic conditions with complete accuracy, our results for area-based SES accord with the pattern of economic growth and regional inequality in China during the reform era [25]. Thus, we think this indicator has some validity. However, a more comprehensive, validated area-based SES measurement is needed that takes into consideration factors such as concentration of poverty, health insurance coverage, proportion of the population with blue collar jobs, unemployment rate, median household income, and median value of owner-occupied houses [47]. Finally, area-based SES was measured at a large geographic scale in our study; it would be instructive to study area-based SES differences among smaller geographic areas in a future study.

## Conclusion

In summary, the effects of area-based SES are complex and multidimensional. Women in lower SES areas may be more likely to ignore symptoms for a variety of economic, social, and cultural reasons. Additionally, women in lower SES areas tend to be less educated and may not understand disease progression, and so may not seek treatment until breast cancer has developed into a late stage. WHO's national cancer control programmes suggest that research to determine the most cost-effective cancer control strategies is especially relevant in developing countries, perhaps even more so than in industrialized countries. However, the available research data for effective and efficient cancer control decision-making are extremely limited in these countries. Our results suggest that in China and other developing countries with similar socioeconomic conditions, breast cancer control programs should focus on ensuring adequate access to screening and improving breast cancer awareness and education. This would facilitate diagnosis at an early stage, particularly for populations living in socioeconomically disadvantaged areas, and would in turn decrease breast cancer mortality and improve surviving patients' quality of life.

## Abbreviations

SES: Area-based socioeconomic status; SEIs: Socioeconomic indicators; SEER: Surveillance Epidemiology and End Results; HSE: percentage Percentage of health-service expenditure in the regional/provincial public affairs general budget; PU/PR: ratio Ratio of urban to rural population; FPI: percentage Percentage of illiteracy in females aged 15 and over; CICAMS: Chinese Academy of Medical Sciences; AJCC: American Joint Committee on Cancer; ER: Estrogen receptor; PR: Progestin receptor; Her-2: Human epidermal growth factor receptor 2; CRF: Case report form; CBE: Clinical breast examination; MRI: Magnetic resonance imaging; BHGI: Breast Health Global Initiative.

## Competing interests

The authors declare that they have no competing interests.

## Authors' contributions

QW helped to analyze, and interpret the data. She also drafted the initial manuscript. JL helped to design the study and interpret the data. SZ helped design the CRF to collect the data. JYL was the local PI who helped with data collection and performed critical revisions of the manuscript. JHF, YP, and RH helped with the data management and analysis. BNZ helped to design the study and is the clinical PI of the study. BZ, HJY, XMX, ZHT, HL, and JJH were the local PIs who helped with data collection. YLQ was the PI of this study and assisted in designing the study and performed critical revisions of the manuscript. All authors read and approved the final manuscript.

## Pre-publication history

The pre-publication history for this paper can be accessed here:

http://www.biomedcentral.com/1471-2407/12/122/prepub
